# Correspondence

**Published:** 1857-01

**Authors:** C. A. Harris, P. H. Austen

**Affiliations:** Baltimore


### CORRESPONDENCE.

BALTIMORE, *December* 29*th*, 1856.

PROF. P. H. AUSTEN:

MY DEAR DOCTOR: Knowing that you have had some experience in the method of mounting teeth recently introduced by Dr. Blandy, I would be glad to have your opinion as to its advantages, compared with other methods now practiced, and would request from you an answer to the following queries:

1. Is it applicable to all cases requiring the insertion of artificial teeth on plate?

2. Are pieces thus made as secure, serviceable and durable as under other methods: and how do they compare in respect of comfort and cleanliness?

3. Are they capable of being as accurately fitted to the mouth?

4. How is this work as regards beauty, in or out of the mouth?

5. Is more or less time required than for other methods, and what is the relative cost of material?

6. Can this work be readily repaired, in case of accident in the hands of the patient or mistakes and accidents of the operator: are the liabilities to error or accident greater than in other methods?

Any information which you may be able to furnish me will be thankfully received by,

Very sincerely and truly, your friend,

C. A. HARRIS.

BALTIMORE, *January* 5*th*, 1857.

PROF. C. A. HARRIS:

*My Dear Sir*—Recent sickness has thrown upon my hands so much deferred work, that I have been compelled thus to delay my answer to yours of the 29th December, and must now ask you to accept a series of laconic answers.

I had lately had occasion to state my opinion of "Dr. Blandy's recently introduced method" in a short communication to the editors of the Dental News Letter, and again to the editor of the Dental Recorder. I have no desire to recall the opinion there given, and will, therefore, refer you to those papers, as containing answers to most if not all of your questions.

I will, however, very cheerfully give you a brief reply to your queries, seriatim—premising thus much, that in stating "its advantages compared with other methods now practiced," you do not understand me as ignoring the excellences of such methods, because I do not specify them. I have no purpose to lessen the established merit of old practices, or place myself in controversy respecting the worth of more recent ones.

(1.) I think it applicable in *all* such cases.

(2.) The metallic plate admits of *perfect* accuracy of adaptation, hence giving security, service and comfort. The close union of the teeth and plate gives great strength, and in the avoidance of all crevices, renders it very easy for the patient to keep the piece clean. I have made special inquiry respecting the existence of any unpleasant taste: thus far I find none; and in some cases where this work was substituted for silver and 18 carat gold, the patient, unasked, remarked the difference in its favor. I have seen pieces six months in wear that have not tarnished, and have not yet seen any piece (with metal of proper composition) that has changed color in wearing. Electro-gilding is a complete remedy for taste or tarnish, in any case where more extended experience may find it to occur.

(3.) The fit is as absolutely accurate as the "impression" is perfect.

(4.) I know no work *out of* the mouth more beautiful than a set of well carved blocks mounted by this process—as to beauty *in* the mouth; that depends upon the shape, color and arrangement of the teeth, as adapted to the peculiarities of each case, and must vary with the care and artistic skill of the operator. Plate teeth, single gum teeth or block work admit of a high degree of finish by this method.

(5.) In both respects the economy is great. As compared with the usual methods practiced, it requires from $\frac{1}{2}$ to $\frac{1}{8}$th the time: its cost, as regards the plate (the teeth being the same in both cases) is from $\frac{1}{4}$ to 1-40th that of gold, varying with the size of the plate, and the use or disuse of electrotype gilding.

(6.) Repairs are readily made and at no risk to the teeth. In the event of accident to the plate, incorrect impression or articulation, change in shape of the mouth, &c. the *same* blocks or single teeth may be readily remounted, in the same or different position, upon a second plate: and this may be done any number of times. I am not aware of any method giving so perfectly this, at times, very acceptable privilege.

As regards accidents during progress of the work—with a correct impression, and a carefully taken articulation, I should feel no fear of any accident or want of accurate adaptation. Certain well known risks and uncertainties attendant upon swaging, soldering, furnace heat, &c., being here dispensed with, we are not so often called upon to utter anathemas against the depots for sending us such miserable plate and for manufacturing such worthless teeth. There are ways and means to get round all these troublesome risks; still it is very pleasant to sail on an open sea with no "breakers ahead."

Hoping that I may have been so happy as to answer your inquiries in a satisfactory manner,

I remain, very sincerely, yours,

P. H. Austen.

